# Evidence of Time-Dependent Hepatic Cytotoxicity and Mitochondrial Remodelling Induced by Palmitoyl Epigallocatechin Gallate vs. Its Native (Poly)Phenol

**DOI:** 10.3390/molecules30132889

**Published:** 2025-07-07

**Authors:** Concepción Medrano-Padial, Cristina García-Viguera, Raúl Domínguez-Perles, Sonia Medina

**Affiliations:** Laboratorio de Fitoquímica y Alimentos Saludables (LabFAS), CSIC, CEBAS, Caumpus Universitario de Espinardo, Edificio 25, 30100 Murcia, Spain; conmedpad@gmail.com (C.M.-P.); rdperles@cebas.csic.es (R.D.-P.); smescudero@cebas.csic.es (S.M.)

**Keywords:** lipophenols, hepatic cells, apoptosis, mitochondria, electronic microscopy, in vitro

## Abstract

Lipophenols, combining phenolic and lipid characteristics in an amphiphilic molecule, offer unique bioactive properties with therapeutic potential, including anti-inflammatory and anti-oxidant effects. Thus, palmitoyl-epigallocatechin gallate (PEGCG), a lipophilic derivative of the extensively studied (poly)phenol epigallocatechin gallate (EGCG), has been stressed concerning enhanced stability in lipid-rich environments and bioavailability due to improved cellular uptake. Nonetheless, the effect of lipophilic esterification on some cellular processes, particularly at the mitochondrial level, remains underexplored. According to this knowledge gap, the present study uncovered the cytotoxic and mitochondrial effects of PEGCG, in vitro, upon the liver hepatocarcinoma cell line HepG2. The range of determinations developed, including the MTS (3-(4,5-dimethylthiazol-2-yl)-5-(3-carboxymethoxyphenyl)-2-(4-sulfophenyl)-2H-tetrazolium) assay, flow cytometry, and electron microscopy, allowed describing the distinct biological potential for both EGCG and PEGCG. Thus, while EGCG exhibited minimal cytotoxicity and apoptosis induction, PEGCG reduced cell viability dose-dependently at 24 h and triggered significant mitochondrial damage, including fragmentation and cristae loss, at 1 µmol/L. However, at 48 h, PEGCG-treated cells recovered viability and mitochondrial structure, suggesting the activation of adaptive mechanisms for the molecular changes induced by PEGCG. These findings underscore the dynamic interplay between lipophilic catechins and cellular stress responses, offering valuable insights into the PEGCG’s potential as a therapeutic agent and laying a foundation for further exploration of its biological power.

## 1. Introduction

Lipophenols are a distinctive class of bioactive compounds consisting of a phenolic moiety, known for its potent anti-oxidant activity, and a fatty acid that confers lipophilic behaviour. This family of bioactive compounds combines the chemical characteristics of lipids and phenolic substances in an amphiphilic molecule that provides unique physicochemical and biological properties [1]. This dual nature enables lipophenols to interact efficiently with other molecules in both aqueous and lipid environments, making them highly versatile in biological systems. In addition, the biochemical properties conferred by the fatty acid moiety to the phenolic structure entail an enhanced therapeutic potential in processes associated with oxidative stress, such as cardiovascular disorders, neurodegenerative conditions, and certain types of cancer [2].

**Figure 1:**
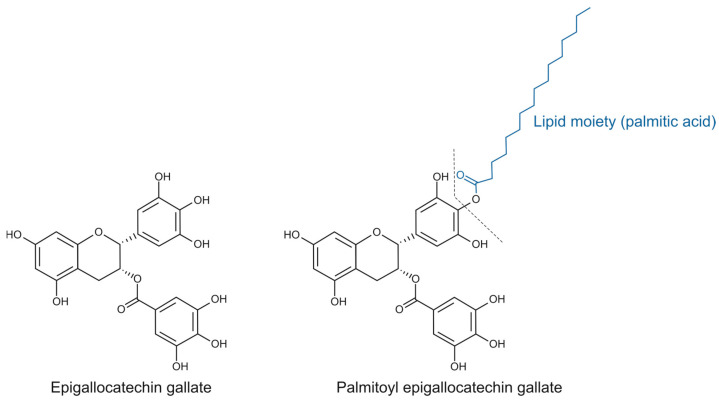
Chemical structure of epigallocatechin gallate (EGCG) and palmitoyl epigallocatechin gallate (PEGCG).

Esterified catechins, such as epigallocatechin gallate (EGCG) ester derivatives, represent a promising category of lipophenolic compounds because of their biological potential [3] (Figure 1). In this aspect, while EGCG has been extensively studied [4], the biological traits of its lipophilic derivative palmitoyl-epigallocatechin gallate (PEGCG), which has garnered attention as a promising bioactive compound due to an enhanced stability in lipid-rich environments and bioavailability due to improved cellular uptake [5], remain underexplored. Notably, the scarce literature on PEGCG reports greater anti-inflammatory activity than EGCG, including stronger inhibition of human cyclooxygenase-2 (COX-2) and subsequent reductions of the related oxylipins [6].

Contrasting the enhanced biological potential demonstrated for PEGCG relative to the native (polyphenol), some concerns have been raised about potential pro-oxidant effects and liver toxicity, especially because these have already been associated with catechins [7]. In this concern, to date, the loss of mitochondrial membrane potential and increased production of reactive oxygen species (ROS) have been specifically reported in preclinical in vivo models [8]. This evidence underscores the importance of mitochondria as critical regulators of cellular energy production and oxidative stress and as potential targets of catechins [9]. In this sense, EGCG has demonstrated a valuable capacity to modulate mitochondrial function [8,10], while the specific effects of PEGCG remain largely unknown. Moreover, detailed studies are needed on its ultrastructural and functional impacts on mitochondria, because of the importance of elucidating the mechanisms behind the referred-to activity. In this regard, it has to be stressed that these cellular effects may also be linked to cell apoptosis—a tightly-regulated form of cell death often initiated by mitochondrial dysfunction—whose dedicated pathways may be activated by catechins, altering the apoptosis/necrosis balance [11].

Based on these antecedents, the present study aims to evaluate the cytotoxic and mitochondrial effects of PEGCG compared to EGCG in hepatocytes (HepG2 cells), a well-recognised in vitro model for liver-related studies. Through cell viability and flow apoptosis assays, as well as mitochondrial ultrastructure analysis via electron microscopy, this research provides new insights into the biological aspects of PEGCG.

## 2. Results and Discussion

### 2.1. Impact of Palmitoyl Epigallocatechin Gallate and Epigallocatechin Gallate on Cell Viability

The hepatocytes’ (HepG2) viability following the treatment with EGCG and PEGCG (from 0.001 to 1.000 μmol/L) was evaluated using the MTS assay, which is based on the mitochondrial conversion of the colourimetric reagent MTS into soluble formazan by dehydrogenase enzymes found only in metabolically active cells.

**Figure 2:**
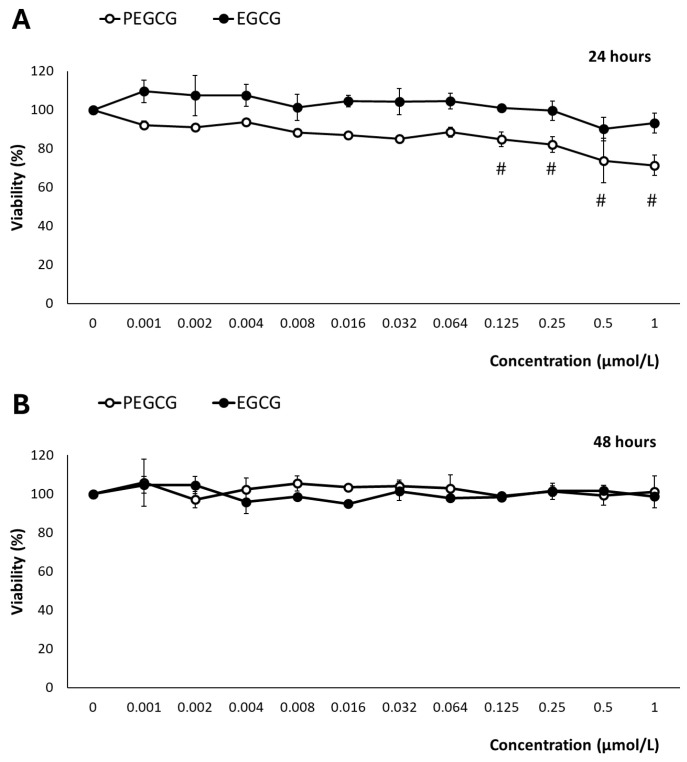
Reduction of tetrazolium salt (MTS) by HepG2 cells exposed for 24 h (**A**) and 48 h (**B**) to 0–1 μmol/mL of EGCG and PEGCG. All values are expressed as mean ± SD (*n* = 3) of viability percentage calculated relative to the untreated control in three independent experiments. Significant differences between given concentration and negative control at *p* < 0.001 (#) according to a paired *t*-test are shown. Data shown are representative of two independent experiments.

After exposure to EGCG, at 24 and 48 h, cell viability remained consistently higher than 90% across all tested concentrations and time points, indicating minimal cytotoxic effects (Figure 2). The impact of EGCG on hepatic cell growth has been widely documented. In contrast to our findings, several studies have described its inhibitory effects through the modulation of various molecular mechanisms that have been attributed to the tumoral character of the HepG2 cell line [12]. In this context, the behaviours of anti-oxidant catechins are complex, as they may exhibit pro-oxidant effects under certain environmental conditions and depending on the concentration [10].

According to the drawbacks presented by the inhibitory effects on hepatocytes’ growth of EGCG and PEGCG, in this study, the concentrations of EGCG and PEGCG evaluated (ranging from 0.001 to 1.000 μmol/L) were selected to set up potential harmful effects within physiologically relevant conditions. These are referred to as the concentrations of bioactive compounds reached in different tissues and cell types after dietary ingestion, gastrointestinal digestion, and intestinal absorption. Such concentrations are for most phytochemicals near 1 μmol/L, as stated by Singh et al. [13]. The main results retrieved offered an accurate representation of these compounds’ biological impact, and were informative about tentative in vivo cytotoxicity. This is essential because previous research on EGCG derivatives has employed supraphysiological doses [14], which may not reflect the true biological relevance of natural compounds, in vivo. Thus, while the highest concentrations assessed can induce cellular responses, they may also cause unintended cytotoxic effects [15], leading to opposite interpretations of their therapeutic potential.

In contrast to EGCG, after 24 h of exposure, PEGCG showed a different pattern, inducing a dose-dependent reduction in cell viability from 0.125 to 1.000 µmol/L (*p* < 0.001), where viability dropped below 80% (Figure 2). The increased cytotoxicity due to the esterification of EGCG can be attributed to the lipophilic nature of PEGCG, which would enhance its cellular uptake and thus greatly facilitate intracellular accumulation. This information suggests that matching doses may lead to a greater intracellular concentration of the lipophilic derivative, higher cellular stress, and, as a final consequence, compromised cell viability [2]. However, by 48 h, this reduction in viability was no longer observed, as cell viability recovered to near 100% across all concentrations. The adaptive mechanisms of HepG2 cells can explain this behaviour in response to PEGCG-induced stress, which is in good agreement with previous studies that have highlighted the capacity of cells to halt apoptosis, contribute to damage repair, and restore normal functions, including the recovery of mitochondrial structure and cellular viability [16,17]. Moreover, the adaptive behaviour of hepatocytes recorded at 48 h, may reflect a balance between the initial stressing effects induced by PEGCG and the activation of protective mechanisms mitigating further damage, such as DNA repair and the regulation of Bcl-2 family proteins involved in apoptosis control [18]. Furthermore, the transient nature of the observed cytotoxicity aligns with the dynamic cellular response to phenolic compounds, which can shift between pro-oxidant and anti-oxidant effects depending on concentration and exposure duration [2].

### 2.2. Cell Death Pathways Analysis: Apoptosis vs. Necrosis

**Figure 3:**
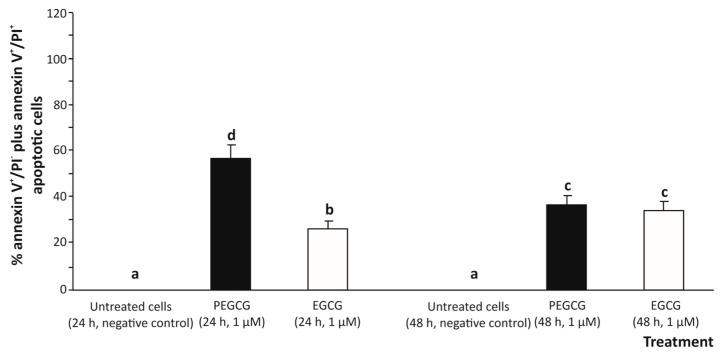
Apoptosis in human hepatocytes (HepG2 cells) exposed to 1 µmol/L epigallocatechin gallate (EGCG) and palmitoyl epigallocatechin gallate (PEGCG) for 24 and 48 h. Bar plots obtained from analysis of the double colour flow cytometry represent percentage of total apoptotic cells (annexin V^+^/PI^−^ plus annexin V^+^/PI^+^). Distinct lowercase letters indicate significantly different values at *p* < 0.05 according to the analysis of variance (ANOVA) and multiple range test of Tukey. Data shown are representative of two independent experiments.

According to an apoptosis assessment using two-colours flow cytometry (annexin V-FITC/PI) (Figure 3), treating cells with 1.000 µmol/L EGCG for 24 and 48 h induced a low-grade apoptotic response determined by the sum of early apoptosis (annexin V^+^/IP^−^) and late apoptosis (annexin V^+^/PI^+^) responses [19] in hepatic (HepG2) cells (26.5% and 34.9%, respectively), compared to the untreated control (undetected).

The significant growth of the apoptotic death percentage induced by EGCG could be due, to some extent, to the pro-oxidant effect of EGCG, particularly through ROS generation, which would play a central role as an apoptosis inducer (secondary to oxidative stress). Indeed, this activity has already been demonstrated when exposing a range of tumoral cell lines to EGCG, which exhibited a cytotoxic effect in a dose-dependent manner [20]. However, under the experimental conditions of this study, at the concentration tested, EGCG cooperates with the anti-oxidant defence of cells and may mitigate ROS-induced damage, leading to a lower apoptotic response.

On the other hand, treating HepG2 cells with 1 µmol/L PEGCG raised the apoptotic death of hepatocytes (up to 47.6%, on average), especially at 24 h of exposure (57.8%), thus exhibiting a higher pro-apoptotic activity for the lipophenolic derivative compared to the negative control (almost undetected) and cells treated with EGCG (30.7%, on average) (Figure 3).

The different pro-apoptotic activity recorded for EGCG and PEGCG may be associated with the biochemical properties conferred to PEGCG by the lipid (palmitic acid) moiety that enhances the lipophilicity of the molecule. This modification would allow it to integrate more efficiently into cellular membranes and achieve higher intracellular concentrations [2]. Hence, the enhanced cellular uptake conferred by the palmitoyl esterification could intensify EGCG’s capacity to interact with key molecular targets, leading to a more pronounced biological effect, already noted in the cytotoxicity study described above.

However, at 48 h, the apoptotic response in PEGCG-treated cells decreased significantly to 37.4% compared to the percentage recorded at 24 h (57.8%), suggesting that hepatic cells can recover from the initial apoptotic signal after metabolising the bioactive molecule. In this context, it has been described that apoptosis, typically considered an irreversible process, can be reversed under certain conditions by a process referred to as anastasis, as previously reported for the cell line HepG2 and mouse primary liver cells [21]. Thus, while minimal data exist on the specific role of lipophenols (such as PEGCG) in this process, the findings in the present work suggest a complex interplay between apoptosis induction and cell survival mechanisms that will require further investigation.

### 2.3. Ultrastructural Changes in Mitochondria

**Figure 4:**
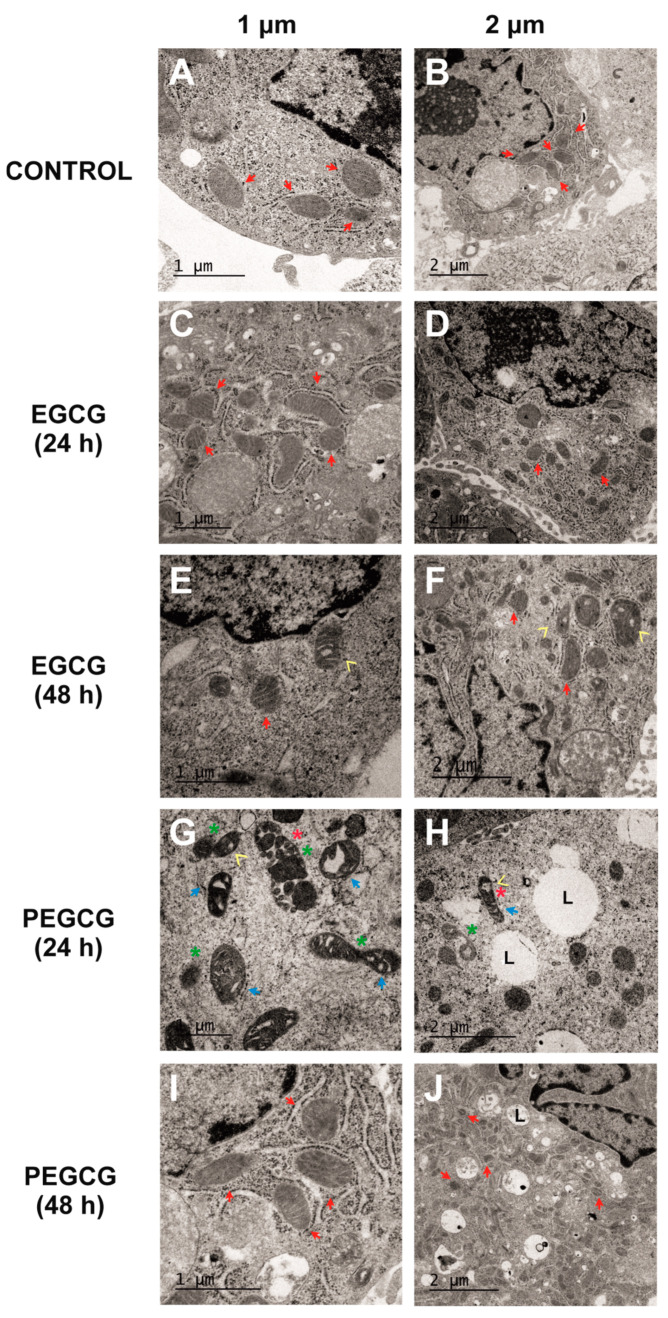
Electron microscopy evidence of changes in HepG2 cells’ ultrastructure after treatment with control culture (**A**,**B**), 1 µmol/L of EGCG during 24 h (**C**,**D**), 1 µmol/L of EGCG during 48 h (**E**,**F**), 1 µmol/L of PEGCG during 24 h (**G**,**H**), and 1 µmol/L of PEGCG during 48 h (**I**,**J**). Control cultures and cells exposed to EGCG displayed normal mitochondrial morphology, with well-defined cristae (red arrow). HepG2 cells exposed to PEGCG showed significant mitochondrial fragmentation (green star), disorganisation, loss of cristae integrity (blue arrow), vacuolisation within mitochondria (yellow arrowhead), and condensed membranes (red star). Some treatments induced the presence of lipid drops (L). Data shown are representative of two independent experiments.

To date, data on ultrastructural changes in mitochondria caused by catechins remain scarce. Thus, while most studies focus on functional elements, such as the loss of mitochondrial membrane potential, rather than detailed morphological alterations, lipophenols are even less explored regarding their impact on mitochondrial ultrastructure. In this sense, TEM was performed to complement the main results retrieved from viability assays and thus gain further insights into the cellular response to EGCG and PEGCG, focused on mitochondrial structure and cellular morphology. Representative images were obtained under the following conditions: untreated cells (negative control, Figure 4A,B), cells treated with EGCG (1 µmol/L) at 24 (Figure 4C,D) and 48 h (Figure 4E,F), and cells treated with PEGCG (1 µmol/L) at 24 (Figure 4G,H) and 48 h (Figure 4I,J).

The assessment of the control cultures (Figure 4A,B) provided images of the normal mitochondrial and HepG2 cells’ morphology, characterised by well-defined cristae (indicated by red arrows) and intact outer membranes. These features indicate preserved structural integrity and are associated with normal mitochondrial function [22].

The HepG2 cells treated with EGCG at 1 µmol/L showed minimal mitochondrial disruption at 24 and 48 h, with most mitochondria retaining their classical morphology (Figure 4C–F). The main ultrastructural changes caused by EGCG consisted of occasional vacuolisation at 48 h, but no significant fragmentation or loss of cristae integrity was recorded. These findings align with the high cell viability observed in MTS assays described before, suggesting that EGCG exerts minimal mitochondrial stress at the tested concentration and time points. Although catechins, including EGCG, have been implicated in mitochondrial-dependent apoptosis and mitochondrial membrane potential loss, the results obtained in the present work indicate that these effects appeared to be dose-dependent [23].

Alternatively, the cells treated with PEGCG at 1 µmol/L for 24 h exhibited significant ultrastructural alterations (Figure 4G,H) consisting of pronounced mitochondrial fragmentation accompanied by a loss of cristae integrity, condensed mitochondrial membranes, and areas of vacuolisation within both the mitochondria and the cytoplasm. While these morphological changes were evident in a large proportion of cells, not all mitochondria were affected, reflecting a heterogeneous cellular response to PEGCG treatment.

Mitochondrial fragmentation is a hallmark of apoptotic pathways and has been shown to amplify the molecular cascade featuring this cell death type by disrupting mitochondrial homeostasis [24]. Hence, the observed damage suggests that PEGCG induces apoptosis through mechanisms similar to those previously described for other catechins, potentially involving mitochondrial permeability transition pore formation, the disruption of mitochondrial membrane potential, and cytochrome c release, all of which lead to the activation of caspases, thus triggering the apoptotic process [8,25].

Interestingly, 48 h post-treatment, mitochondrial structure in the PEGCG-treated cells showed substantial recovery, with many mitochondria regaining their morphology and cristae organisation (Figure 4I,J). As referred to before, this recovery can be explained by the capacity of hepatic cells to initiate the adaptive process known as anastasis, wherein cells reverse early apoptotic changes to survive [21]. Incomplete mitochondrial outer membrane permeabilisation may allow residual mitochondria to retain partial functionality, providing the energy necessary for recovery processes like mitophagy and mitochondrial biogenesis. Using these mechanisms, hepatocytes restore structural integrity and support bioenergetic functions. Moreover, fragmented mitochondria are known to undergo fusion, promoting metabolic regeneration during recovery [16]. In this regard, a previous study evidenced that catechins can promote the recovery of mitochondrial structure and function in cells subjected to oxidative stress induced by simulated diabetes by augmenting the levels of indicators associated with mitochondrial biogenesis through eNOS activation in in vitro and in vivo models [26]. Against this background, this study provides new perspectives into how amphiphilic molecules, such as PEGCG, may play an important role in the adaptation of cells with mitochondrial dysfunction, which could enhance treatment response, with subsequent potential benefits in a wide range of chronic and degenerative diseases.

## 3. Materials and Methods

### 3.1. Chemicals and Reagents

Trypsin-ethylenediaminetetraacetic acid, Eagle’s Minimum Essential Medium (EMEM), L-glutamine, fetal bovine serum (FBS), penicillin/streptomycin, and essential amino acids were obtained from Gibco (ThermoFisher Scientific, Madrid, Spain), and the 24-well plates from Corning (New York, NY, USA). Uranyl acetate was obtained from Fisher (ThermoFisher Scientific, Madrid, Spain). Trypsin, (3-(4,5-dimethylthiazol-2-yl)-5-(3-carboxymethoxyphenyl)-2-(4-sulfophenyl)-2H-tetrazolium (MTS) salt, glutaraldehyde, osmium tetroxide, cacodylate buffer solution, propylene oxide, Epon resin, lead citrate, and ethylenediaminetetraacetic acid (EDTA) were from Sigma-Aldrich (Steinheim, Germany). The apoptosis detection kit, including Annexin V-fluoresceine isothiocyanate (FITC) and propidium iodide (PI) reagents (reference number ab14085), was from Abcam (Abcam, Cambridge, UK).

### 3.2. Cell Line and Culture Conditions

The HepG2 cell line (HB-8065) from human liver carcinoma was obtained from the American Type Culture Collection (ATCC, Rockville, MD, USA). The cell line HepG2 was cultured in EMEM containing 10% FBS and 1% penicillin-streptomycin, maintained at 37 °C with 5% CO_2_. The number of passages of the HepG2 cells used in this study ranged from 15 to 20. Cells were grown until 80% confluence in 75-cm^2^ plastic flasks, detached with trypsin-EDTA at 0.25%, and subcultured at 1:2.

### 3.3. Cytotoxicity Assay

For cytotoxicity studies, HepG2 cells were plated in 96-well plates at a density of 5 × 10^4^ cells per well. After 24 h, cells were treated with varying concentrations of EGCG and PEGCG (from 0.001 to 1.000 μmol/L) for 24 and 48 h. A culture medium without the extract was used as a control group. Concerning the settings of the cytotoxicity assay, a solvent control (0.5% of DMSO) was included. EGCG was taken as a reference control compound to establish the cytotoxicity of its esterified form with palmitic acid (PEGCG). Cell viability was measured using a CellTiter 96^®^ Aqueous One Solution Cell Proliferation Assay (Promega, Madison, WI, USA) as described by Medrano-Padial et al. [27].

### 3.4. Double-Colour Flow Cytometry Analysis of Cell Apoptosis: Evaluation of Annexin-v-FITC/Propidium Iodide

Following the results obtained in the cytotoxicity assay, HepG2 cells were treated with 1 µmol/L of EGCG or PEGCG for 24 or 48 h. Control cells were left untreated. After exposure to PEGCG or EGCG for the time specified, cells were harvested and washed twice with cold PBS. Apoptosis was assessed according to the methodology described by Domínguez-Perles et al. [28]. Briefly, after experimental treatments, cells were stained with annexin V-FITC and propidium iodide (PI). HepG2 cells (3.0 × 10^5^) were incubated with 200 μL of annexin-V-FITC diluted 1:200 in a binding buffer (10 mM HEPES/NaOH, (pH 7.4), 150.0 mM NaCl, 5.0 mM KCl, 5.0 mM MgCl_2_, and 1.8 mM CaCl_2_), and remained at room temperature and protected from light for 20 min. After incubation and immediately before flow cytometry analysis, 5 μL of 50 μg/mL PI was added. Then, stained cells were analysed using a BD FACSCalibur Flow Cytometer (Becton Dickinson, San Jose, CA, USA). A total of 5000 events were collected per sample. Data were analysed using FlowJo^TM^ v10.10 software (Becton Dickinson, CA, USA), and the percentages of apoptotic cells after each separate treatment were determined.

### 3.5. Transmission Electron Microscopic Ultrastructure Analysis of EGCG and PEGCG-Treated HepG2 Cells

Cultured hepatic cells were exposed to 1 µmol/L EGCG or PEGCG for 24 or 48 h. Transmission electron microscopy (TEM) was utilised to evaluate the impact of the selected catechin and its lipid derivative on mitochondrial ultrastructure. Sample preparation was done according to the procedure described by Medrano-Padial et al. [29], with minor modifications. Briefly, cells were fixed in 2.5% glutaraldehyde for 30 min, followed by a post-fixation in 1% osmium tetroxide for 2.5 h. Then, they were washed in cacodylate buffer solution and treated with uranyl acetate for 2 h, as referred to in the literature [30]. Dehydration was carried out through a graded series of ethanol (30%, 50%, 70%, 90%, and 100%). Following this process, cells were treated twice with propylene oxide, embedded in Epon resin, and sectioned to a thickness of 60 nm using a Leica UC6 ultramicrotome (Wetzlar, Germany). Finally, the ultrathin sections were double-stained with uranyl acetate and lead citrate and analysed using a Jeol 1011 TEM (Jeol, Tokyo, Japan) at 80 kV.

### 3.6. Statistical Analysis

All assessments were performed on two independent experiments, each of them including three replicates (*n* = 3), and were presented as the mean ± standard deviation (SD). Statistical evaluations were carried out, for each independent experiment, at a significance level of 5% using the SPSS 29.0 software package (LEAD Technologies, Inc., Chicago, IL, USA). A one-way analysis of variance (ANOVA) was applied to the data, following verification that the assumptions for ANOVA—normality of residuals and homogeneity of variances—were satisfied using the Kolmogorov–Smirnov and Levene tests, respectively. When significant differences were detected, the different variables were compared using Tukey’s multiple range test.

## 4. Conclusions

This study provides new insights into the effects of EGCG and its lipophilic derivative PEGCG on HepG2 cells, focusing on cell viability, apoptosis, and mitochondrial ultrastructure. While EGCG exhibited minimal cytotoxicity and apoptotic activity at physiologically relevant concentrations, PEGCG demonstrated a more pronounced biological response, particularly at 24 h. This difference reinforces the central relevance of the amphiphilic trait conferred by the lipid moiety to catechins, which entails enhanced cellular uptake and interaction with intracellular targets. This fact should be considered in both bioactivity and cytotoxic effects, which indicates the necessity of setting up the actual concentration of lipophenols associated with each of these biological effects. Moreover, the transient nature of PEGCG’s cytotoxic effects and mitochondrial damage suggests the capacity of adaptive mechanisms in hepatocytes (such as anastasis) to restore normal cell morphology and functions, highlighting the dynamic balance between apoptosis induction and recovery. The relevance of the findings described in the present work encourages the development of complementary studies providing quantitative data on mitochondrial shape, size, or subtype characterisation to further understand the cytotoxic effect of PEGCG at the ultrastructural level in hepatocytes. In addition, the constraints associated with in vitro research would need in vivo confirmation that would provide a consistent validation of the main results presented in this work. Thus, complementing the information provided in the present work on the cytotoxicity of lipophenols would lead to exploring these amphiphilic molecules for potential therapeutic applications and lay the groundwork for future investigations into their accurate operative concentration and mechanisms of action.

## Data Availability

Data are contained within the article.

## Abbreviations

The following abbreviations are used in this manuscript:

ANOVA	Analysis of variance
COX-2	Cyclooxygenase-2
EGCG	Epigallocatechin gallate
EMEM	Eagle’s Minimum Essential Medium
EDTA	Ethylenediaminetetraacetic acid
FBS	Fetal bovine serum
FITC	Fluoresceine isothiocyanate
HepG2	Liver hepatocarcinoma cell line
MTS	((3-(4,5-dimethylthiazol-2-yl)-5-(3-carboxymethoxyphenyl)-2-(4-sulfophenyl)-2H-tetrazolium salt)
PEGCG	Palmitoyl-epigallocatechin gallate
PI	Propidium iodide
ROS	Reactive oxygen species
SD	Standard deviation
TEM	Transmission electron microscopy
